# Contributory factors to reporting distance as a barrier to health facility visit among reproductive-age Senegalese women: A survival analysis

**DOI:** 10.1371/journal.pone.0321850

**Published:** 2025-04-16

**Authors:** Michael Ekholuenetale

**Affiliations:** Faculty of Science and Health, School of Health and Care Professions, University of Portsmouth, Hampshire, United Kingdom; Aix-Marseille Universite, FRANCE

## Abstract

**Background:**

The burden of distance to healthcare facility is a factor of maternal morbidity and mortality in resource-constrained settings. In Senegal, little is known about distance or travel time as a barrier to health facility visit. The objective of this study was to assess distance as a barrier in reaching a health facility and its contributory factors among Senegalese women.

**Methods:**

The data from 2023 Senegal Demographic and Health Survey (SDHS) was used in this study. A total sample of 16,583 women aged 15–49 years was analysed. Kaplan-Meier plot was used to estimate the median time to healthcare facilities. Furthermore, the factors of distance as a barrier in reaching a health facility were examined using multivariable Cox regression and reported as adjusted hazard ratio (aHR) with its 95% confidence intervals (CI). The statistical significance was determined at p < 0.05.

**Results:**

Overall, 35.8% of women reported distance as a barrier to reaching a health facility. The median time to a health facility was 13 minutes. Approximately two-thirds of the women reported walking to the nearest healthcare facility (66.4%), while others used animal-drawn cart (12.5%), motorcycle/scooter (9.3%) and car/truck (7.8%) respectively. Poular, Serer and Mandingue women had 10% (aHR= 0.90; 95% CI: 0.84–0.98), 19% (aHR= 0.81; 95% CI: 0.74–0.90) and 14% (aHR= 0.86; 95% CI: 0.76–0.97) reduction in the risk of reporting distance as a barrier in reaching a health facility, when compared with Wolof women. In addition, women from Dakar, Ziguinchor, Saint-Louis, Tambacounda, Kaolack, Thiès, Louga, Fatick, Kolda, Matam, Kaffrine, Kedougou and Sedhiou had higher risk of reporting distance as a barrier in reaching a health facility, when compared with women from Diourbel region. Rural women had 36% higher risk of reporting distance as a barrier in reaching a health facility (aHR= 1.36; 95% CI: 1.27–1.44), when compared with their urban counterparts.

**Conclusion:**

Based on the findings of this study, rural and remote women have distance as a barrier to health facility visit. The travel time to a health facility is a critical indicator of the burden of distance to health facility and can therefore better inform health services planning for people residing in rural and remote locations in Senegal.

## Introduction

Maternal mortality remains a significant public health challenge in several sub-Saharan African countries (SSA) [1]. According to the World Health Organization (WHO), timely access to healthcare facilities is crucial for preventing and managing pregnancy-related complications [2]. Maternal health is a crucial subject matter for today and the future and must be given attention in global health initiatives [3]. The first target of the third Sustainable Development Goal (SDG 3.1) is to decrease maternal mortality to less than 70 deaths per 100,000 live births by 2030 [4]. However, by 2020, Senegal had a maternal mortality ratio (MMR) of 261 deaths per 100,000 live births from 638 deaths per 100,000 live births in 2000. This shows an average annual rate of reduction (ARR) of 4.7%, with overall reduction of 60.7% in MMR between 2000 and 2020 [5]. To achieve reduction in maternal deaths, substantial and concerted efforts must be made to remove a variety of barriers to the use of maternal health services, including the burden of distance to healthcare facility.

Access to timely health care service utilisation is a critical factor influencing the health outcomes of individuals, particularly among reproductive age women. In Senegal, like many other developing countries, delays in seeking and accessing healthcare can significantly impede maternal and child health. Understanding the factors associated with distance as a barrier in reaching a health facility among reproductive age women is essential for designing interventions aimed at improving maternal health outcomes. There has been no work conducted among women of reproductive age in Senegal to estimate the travel time or distance as a barrier to health facility visit. However, a previous study conducted among 45,945 women in Namibia, Burkina Faso, Cote D’Ivoire, Kenya, and Lesotho revealed about 30% of the women reported distance as a barrier in reaching a health facility [6]. Furthermore, travel delays have been reported as one of the three delays in getting health care [7]. Poor roads infrastructure, topography; such as rivers and mountains as well as the distance to healthcare facilities all contribute to delays in getting to health facility [8].

Geographic accessibility is a critical factor influencing the timing to healthcare facility. In rural areas, healthcare facilities are often sparse and distant, requiring women to travel long distances to access care [9]. Poor road infrastructure and lack of reliable transportation further compound this challenge [10]. Women living in remote areas may face significant delays in reaching healthcare facilities, which can adversely affect their health outcomes [11]. The burden of distance to health facility remains a major contributor to maternal mortality and morbidity in resource-constrained settings, including Senegal [12,13]. Transportation to healthcare facilities in Senegal often involves navigating a mix of urban and rural challenges. In urban areas like Dakar, patients commonly use public buses, taxis, or ride-hailing services where traffic congestion can cause delays. In rural regions, where healthcare facilities are more dispersed, access is more difficult. Many people rely on motorbikes, horse-drawn carts, or walking, which can be time-consuming and physically demanding. Limited transport options remain a significant barrier to timely healthcare access for many women in resource-poor settings including Senegal [14].

Notably, there is dearth of data about the travel time to health facility and the contributory factors of distance as a barrier in reaching a health facility in Senegal. The objective of this study was to assess distance as a barrier to health facility among Senegalese women.

## Methods

### Data source

Data from the 2023 Senegal Demographic and Health Survey (SDHS) was used in this study. A total sample of 16,583 women aged 15–49 years was analysed. The data collection was carried out from January to August 2023 with a break of approximately two months, making a period of five months of data collection. The individual women’s questionnaire was used to capture information from women aged 15–49 years. The questionnaire asked questions on socio-demographic characteristics of the respondent; reproductive health; contraceptive use; pregnancy and postnatal care; fertility preferences; and maternal mortality.

### Sampling design

The Continuous SDHS 2023 targets: women aged 15–49 years, men aged 15–59 years and children aged 0–59 months. The scope of the survey covers the entire territory and concerns a stratified national sample of approximately 8,800 households, drawn at two stages. This sample makes it possible to produce representative results at the regional level. To constitute the sample, 400 enumeration areas (186 in urban areas and 214 in rural areas) were selected at the first stage of sampling by carrying out a systematic selection with probability proportional to size. A count of households in each of these enumeration areas provided the list of households from which a second-stage sample of 22 households was drawn with a systematic selection with equal probability. All women aged 15–49, usual residents or visitors, identified in these households were individually surveyed.

### Variables selection and measurement

#### Outcome variable.

The main outcome variable in this study was reporting distance as a barrier to health facility. It was derived from the question; “Getting medical help for self: distance to health facility” and responses were coded as “1” if a respondent reported as “Big problem” or “0” if a respondent reported “Not a big problem”.

In addition, the timing to healthcare facility was also utilised, and derived from the question; “Minutes to nearest healthcare facility”. This was used in modelling the Cox proportional hazard regression.

The mode of transportation was derived from the question; “Mode of transportation to nearest healthcare facility” in the SDHS individual woman dataset.

#### Explanatory variables.

The factors examined in this study are based on previous studies and presented in Table 1 below [15–18].

**Table 1:** Categories and operational definition of independent variables.

	Variables	Definitions
1.	Age (in years)	Age of the respondent (15–24, 25–34, 35+)
2.	Religion	This is the religious practice of respondents (Muslims, Christians, Others)
3.	Ethnicity	Wolof, Poular, Serer, Mandingue, Diola, Soninke, Foreign ethnicity, Other
4.	Region	Dakar, Ziguinchor, Diourbel, Saint-Louis, Tambacounda, Kaolack, Thiès, Louga, Fatick, Kolda, Matam, Kaffrine, Kedougou, Sedhiou
5.	Place of residence	Area of residence (urban, rural)
6.	Years lived in residence	<5 years, 5+ years
7.	Education	No education/primary, Secondary/higher
8.	Wealth quintiles*	Economic/wealth status of the household (poor, non-poor)
9.	Marital status	Unmarried, married
10.	Visited health facility last 12 months	Not visited, visited
11.	Health insurance coverage	Insured, uninsured

*For the calculation household wealth status, household assets such as ownership of television, radio, bicycle possessed by the household and housing quality such as type of floor, wall and roof were taken into consideration. Each item is assigned a factor score generated through principal component analysis which are then summed and standardized for the households. These standardised scores places the households in a continuous scale based on relative wealth scores. The scores thus obtained from a continuous scale are subsequently categorised into quintiles to rank the household as poorest/poorer/middle/richer/richest to richest [19]. The poorest and poorer categories were grouped as poor, others (middle, richer, richest) were grouped as non-poor.

### Ethical consideration

In this study, access to the data was granted to the author upon registration and request by MEASURE DHS/ICF. In order to guarantee the privacy of respondents, the MEASURE DHS Programme complies with standard regulations. With regard to the respect for human subjects, ICF International makes sure the survey conforms with the regulations set forth by the US Department of Health and Human Services. This study did not require any ethical approval from the ethics committee of the author’s university. The additional ethical guidelines for the data collection process are available at http://goo.gl/ny8T6X.

### Analytical approach

To calculate the estimates of distance as a barrier in reaching a health facility, Stata survey (‘svy’) module was utilised to account for sampling weights, clustering, and stratification [20,21]. Variance-inflation factor was used to assess multicollinearity, and a value of less than 10 was deemed appropriate [22,23]. Since no variable was found to be interdependent, none was removed from the model. Percentage was used to estimate women reporting distance as a barrier in reaching a health facility and the mode of transportation to nearest health facility. The summary statistics and Kaplan-Meier plot were used to calculate median time to a health facility. Chi-square test was used to examine the association between reporting of distance as a barrier in reaching a health facility and independent variables. Finally, Cox proportional hazard regression models were used to estimate the contributory factors of distance as a barrier in reaching a health facility [15,16]. The Cox estimates were presented as hazard ratios with 95% confidence intervals of model outputs. Statistical signiﬁcance was set at p< 0.05. Stata Version 17 (StataCorp., College Station, TX, USA) was used for data analysis.

The survivor function of the travel time to health facility is the estimate of the probability that a woman’s time to health facility is not a barrier, while the hazard function gives the instantaneous rate per unit time of experiencing barrier with the travel time to health facility, given that the individual women have no barrier with travel time to health facility. The effect of each factor viz-a-viz its levels was presented as hazard ratios (HR) with its confidence intervals. If HR>1, then there is a higher risk of reporting distance as a barrier to a health facility, HR < 1 implies lower risk of reporting distance as a barrier to a health facility while HR = 1 has no effect of reporting distance as a barrier to a health facility. The significant variables in each of the unadjusted Cox regression were used in the adjusted Cox regression to assess association with the outcome variable while controlling for confounders.

### Justification for use of survival analysis

Survival analysis was well-suited for modeling the contributory factors influencing whether distance serves as a barrier to arriving at a health facility, using estimated time to arrival as the key time-to-event variable. The traditional regression methods would not adequately capture the time-dependent nature of access to health facility, whereas survival analysis accounted for both the timing and the right-censoring of data (e.g., women who did not report the time to arrive at health facility). By using survival models such as the Cox proportional hazards model, the probability of arrival at health facility was estimated while adjusting for confounders. This approach provides insights into the differential impact of distance across populations, identifying subgroups that experience greater delays. Findings from survival analysis can guide policymakers in designing interventions that reduce geographic barriers, enhance emergency referral systems, and improve overall health service accessibility, ultimately reducing preventable delays in care.

## Results

Table 2 shows the median time to healthcare facility (13 minutes). Overall, 35.8% of women reported distance as a barrier to reaching a health facility. Christians, Wolof, Serer, Diourbel, Fatick, Keffrine, rural, with at most primary education, poor, married and not visited health facility last 12 months had the longest median time to healthcare facility respectively. In addition, Poular, foreign ethnicity, Saint-Louis, Tambacounda, rural, with at most primary education, poor, married and not visited health facility last 12 months women reported higher proportion of distance as a barrier in reaching a health facility respectively. See Table 2 for the details.

**Table 2:** Distribution of median time and reporting distance as a barrier in reaching a health facility.

Variable	n (%)	Median time (minutes) to healthcare facility	Proportion reporting distance as a barrier in reaching a health facility	P (Chi^2^ test)
**Age**				0.331 (4.26)
15-24	7265 (43.8)	12.0	35.6 (32.9-38.4)	
25-34	4563 (27.5)	14.0	36.9 (34.2-39.8)	
35+	4755 (28.7)	15.0	35.0 (31.9-38.1)	
**Religion**				0.545 (2.28)
Muslims	16051 (96.8)	13.0	35.8 (33.4-38.3)	
Christians	528 (3.2)	15.0	35.5 (29.3-42.1)	
Others	4 (0.0)	8.5	0.0 (0.0-0.0)	
**Ethnicity**				0.002 (132.68)
Wolof	5488 (33.1)	15.0	33.3 (29.6-37.3)	
Poular	5921 (35.7)	11.0	41.5 (37.5-45.6)	
Serer	2001 (12.1)	15.0	35.2 (29.7-41.1)	
Mandingue	1353 (8.2)	10.0	29.2 (24.0-35.1)	
Diola	683 (4.1)	10.0	35.3 (28.0-43.4)	
Soninke	209 (1.3)	10.0	19.8 (10.8-33.5)	
Foreign ethnicity	8 (0.1)	8.5	46.7 (12.0-84.9)	
Other	920 (5.6)	10.0	32.0 (26.0-38.8)	
**Region**				<0.001 (696.94)
Dakar	1413 (8.5)	10.0	27.9 (23.5-32.7)	
Ziguinchor	807 (4.9)	14.0	40.6 (33.8-47.7)	
Diourbel	1282 (7.7)	20.0	27.4 (18.8-38.1)	
Saint-Louis	1155 (7.0)	10.0	58.3 (50.3-65.9)	
Tambacounda	1211 (7.3)	10.0	49.6 (39.4-59.9)	
Kaolack	1117 (6.7)	15.0	38.1 (28.8-48.3)	
Thiès	1531 (9.2)	10.0	26.2 (20.6-32.7)	
Louga	1252 (7.6)	10.0	45.2 (36.0-54.6)	
Fatick	1280 (7.7)	20.0	41.6 (30.7-53.4)	
Kolda	1027 (6.2)	14.0	41.8 (33.3-50.8)	
Matam	1285 (7.8)	10.0	41.4 (30.5-53.3)	
Kaffrine	1257 (7.6)	25.0	42.0 (32.0-52.8)	
Kedougou	867 (5.2)	10.0	39.3 (30.3-49.1)	
Sedhiou	1099 (6.6)	10.0	42.2 (35.7-48.9)	
**Place of residence**				<0.001 (843.97)
Urban	7471 (45.0)	10.0	24.8 (22.0-27.8)	
Rural	9112 (55.0)	15.0	46.4 (42.4-50.5)	
**Years lived in residence**				0.024 (24.08)
<5 years	1872 (11.3)	10.0	31.0 (26.4-36.0)	
5+ years	14711 (88.7)	14.0	36.5 (34.0-39.0)	
**Education**				<0.001 (143.41)
No education/primary	10924 (65.9)	15.0	39.1 (36.2-42.1)	
Secondary/higher	5659 (34.1)	10.0	29.8 (27.2-32.6)	
**Wealth**				<0.001 (294.01)
Poor	8156 (49.2)	15.0	44.3 (41.0-47.6)	
Non-poor	8427 (50.8)	10.0	31.0 (28.3-33.8)	
**Marital status**				<0.001 (62.47)
Unmarried	4753 (28.7)	10.0	31.4 (28.5-34.4)	
Married	11830 (71.3)	15.0	37.7 (35.1-40.5)	
**Visited health facility last 12 months**				0.012 (16.59)
Not visited	7680 (46.3)	15.0	37.6 (34.7-40.5)	
Visited	8903 (53.7)	11.0	34.5 (32.0-37.1)	
**Health insurance coverage**				<0.001 (42.53)
Not covered	15190 (91.6)	13.0	36.5 (34.0-39.0)	
Covered	1393 (8.4)	14.0	27.5 (23.0-32.4)	
**Total estimates**	16583 (100.0)	13.0	35.8 (33.4-38.3)	

P Chi-square test in the proportion of reporting that distance is a barrier in accessing health facilities.

***n*** sample size.

**Fig 1:**
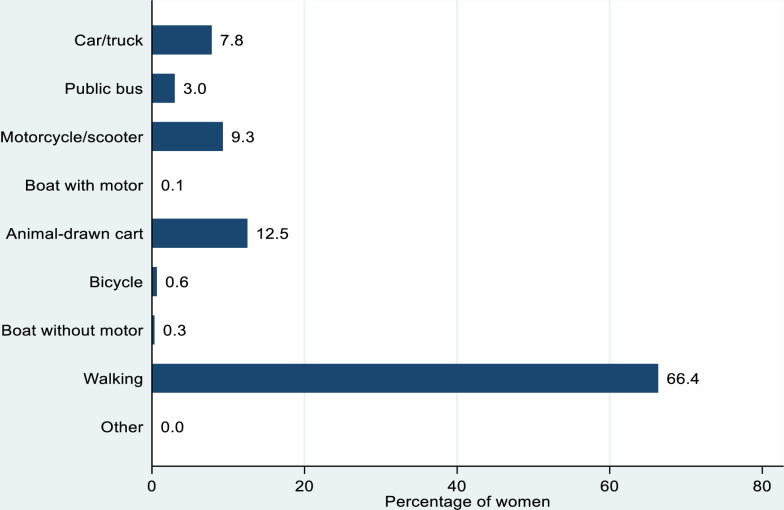
Mode of transportation to nearest healthcare facility.

Fig 1 shows the mode of transportation by women to the nearest healthcare facility. Approximately two-thirds of the women reported walking to the nearest healthcare facility (66.4%). While others used animal-drawn cart (12.5%), motorcycle/scooter (9.3%) and car/truck (7.8%) respectively.

Table 3 shows the adjusted hazard ratio estimates of the contributory factors of distance as a barrier in reaching a health facility. All the factors were examined in the unadjusted Cox hazard model since no variable was found to be interdependent. Poular, Serer and Mandingue women had 10% (aHR= 0.90; 95% CI: 0.84–0.98), 19% (aHR= 0.81; 95% CI: 0.74–0.90) and 14% (aHR= 0.86; 95% CI: 0.76–0.97) reduction in the risk of reporting distance as a barrier in reaching a health facility, when compared with Wolof women. In addition, women from Dakar, Ziguinchor, Saint-Louis, Tambacounda, Kaolack, Thiès, Louga, Fatick, Kolda, Matam, Kaffrine, Kedougou and Sedhiou had higher risk of reporting distance as a barrier in reaching a health facility, when compared with women from Diourbel region. Rural women had 36% higher risk of reporting distance as a barrier in reaching a health facility (aHR= 1.36; 95% CI: 1.27–1.44), when compared with their urban counterparts.

**Table 3:** Hazard ratio of contributory factors to distance as a barrier in reaching a health facility.

Variable	Unadjusted Hazard Ratio	95% Confidence Interval	Adjusted Hazard Ratio	95% Confidence Interval
**Age**				
15-24	1.00			
25-34	0.99	0.94-1.06		
35+	1.01	0.94-1.07		
**Religion**				
Muslims	1.00			
Christians	1.04	0.91-1.19		
Others	–			
**Ethnicity**				
Wolof	1.00		1.00	
Poular	1.34	1.25-1.42	0.90	0.84-0.98
Serer	0.82	0.75-0.89	0.81	0.74-0.90
Mandingue	1.21	1.09-1.35	0.86	0.76-0.97
Diola	1.45	1.26-1.66	0.99	0.84-1.18
Soninke	0.99	0.75-1.33	0.76	0.57-1.01
Foreign ethnicity	2.45	0.79-7.62	2.52	0.81-7.84
Other	1.45	1.29-1.63	1.08	0.95-1.23
**Region**				
Dakar	2.39	2.06-2.76	3.01	2.58-3.52
Ziguinchor	2.48	2.13-2.90	2.70	2.24-3.24
Diourbel	1.00		1.00	
Saint-Louis	2.80	2.45-3.20	3.01	2.62-3.47
Tambacounda	3.34	2.93-3.82	3.70	3.20-4.28
Kaolack	1.79	1.54-2.08	1.90	1.64-2.21
Thiès	1.88	1.62-2.18	2.08	1.80-2.42
Louga	2.50	2.18-2.86	2.52	2.20-2.90
Fatick	1.36	1.19-1.55	1.48	1.29-1.71
Kolda	2.26	1.95-2.61	2.44	2.08-2.85
Matam	2.67	2.33-3.05	2.86	2.46-3.33
Kaffrine	1.27	1.10-1.45	1.26	1.10-1.45
Kedougou	2.18	1.87-2.54	2.38	2.01-2.83
Sedhiou	2.59	2.24-2.97	2.67	2.27-3.13
**Place of residence**				
Urban	1.00		1.00	
Rural	1.16	1.09-1.22	1.36	1.27-1.44
**Years lived in residence**				
<5 years	1.00			
5+ years	1.03	0.95-1.12		
**Education**				
No education/primary	1.00			
Secondary/higher	0.96	0.91-1.02		
**Wealth**				
Poor	1.00		1.00	
Non-poor	0.91	0.86-0.95	1.01	0.95-1.07
**Marital status**				
Unmarried	1.00			
Married	0.98	0.93-1.04		
**Visited health facility last 12 months**				
Not visited	1.00			
Visited	0.95	0.91-1.01		
**Health insurance coverage**				
Not covered	1.00		1.00	
Covered	0.78	0.70-0.86	0.96	0.86-1.06

**Fig 2:**
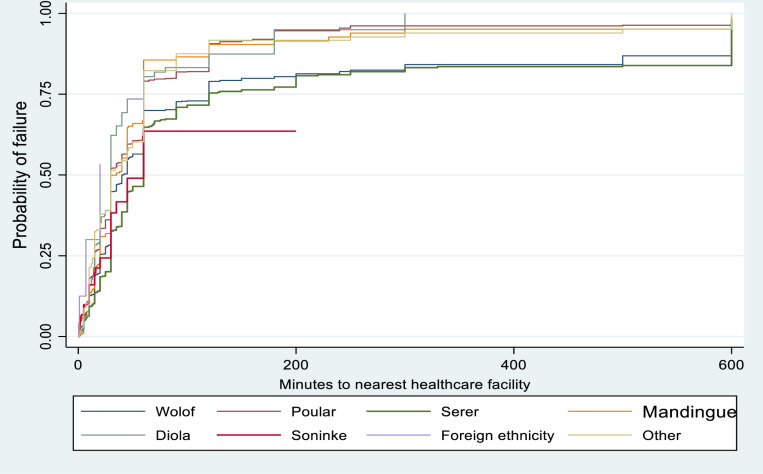
Kaplan-Meier failure estimates of time to healthcare facility by ethnicity.

Fig 2 showed the Kaplan-Meier failure estimates of median time (minutes) to nearest healthcare facility by ethnicity. Overall, there were disparities in the timing to nearest healthcare facilities in Senegal across different ethnic groups. Serer and Soninke have higher median time to nearest health facilities.

**Fig 3:**
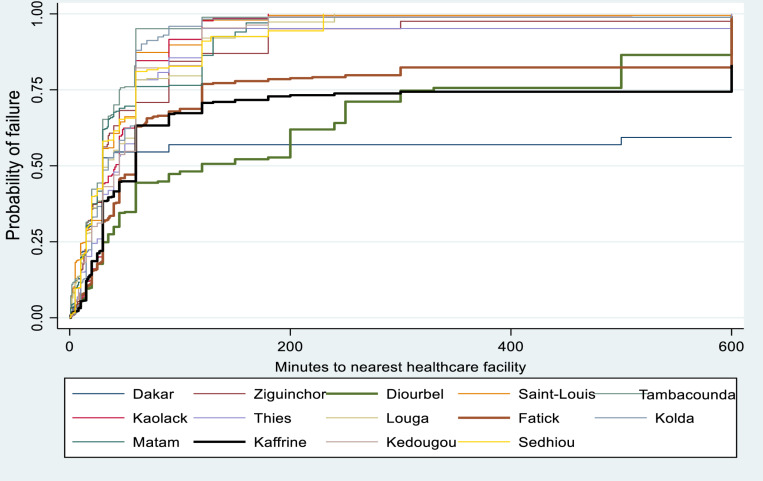
Kaplan-Meier failure estimates of time to healthcare facility by region.

Fig 3 showed the Kaplan-Meier failure estimates of median time (minutes) to nearest healthcare facility by geographical region. Overall, there were disparities in the timing to nearest healthcare facilities in Senegal across different geographical regions. Diourbel, Kaffrine and Fatick have higher median time to nearest health facilities.

**Fig 4:**
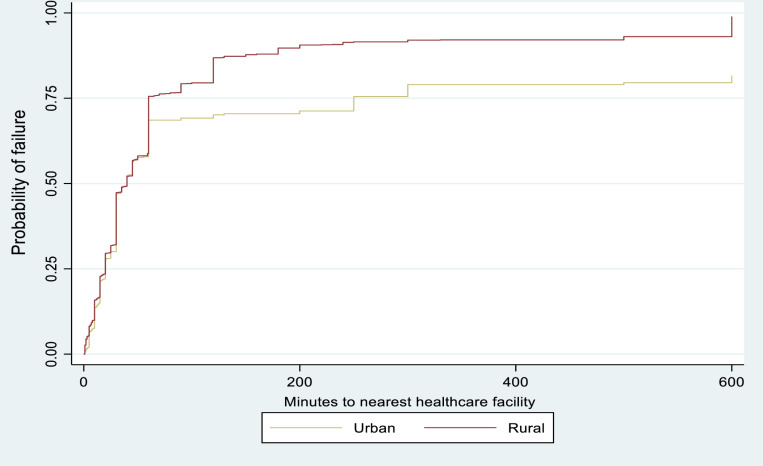
Kaplan-Meier failure estimates of time to healthcare facility by place of residence.

Fig 4 showed the Kaplan-Meier failure estimates of median time (minutes) to nearest healthcare facility by the place of residence. Overall, there were disparities in the timing to nearest healthcare facilities in Senegal across rural and urban residence. The urban and rural residence have no clear difference in median time to nearest health facilities.

S1, S2, S3, S4, S5, S6, S7 and S8 Figs showed the Kaplan-Meier failure estimates of time (minutes) to nearest healthcare facility by women’s age, religion, family motility, education, household wealth, marital status, visit to health facility within the previous 12 months and health insurance respectively. These show little disparities across the categories of various factors. See appendix for the details.

## Discussion

This is the first study to estimate the travel time to healthcare facility and examine the contributory factors to reporting distance as a barrier in reaching a health facility among reproductive age women in Senegal. One of the three delays found to be a significant factor in maternal mortality and morbidity in underdeveloped countries is delay in reaching health facilities [24]. Approximately two-thirds walk to health facilities. From previous studies, the means of having to walk to health facilities [25,26], did not address the accessibility need, as the journey would be lengthened and cumbersome. In Senegal, especially in rural areas, there is a dearth of data on the burden of distance to health facilities and its contributory factors. The findings from this study revealed that over one-thirds (35.8%) of women reported distance as a barrier to healthcare facilities. This is consistent with the findings from a previous study involving women from five SSA countries (Namibia, Burkina Faso, Cote D’Ivoire, Kenya, and Lesotho) where 29.4% of the women reported distance as a barrier in reaching a health facility [6]. The median travel time to reach the nearest healthcare facility was found to be 13 minutes. This is consistent with the findings from Senegal of average travel time to reach health facilities [27]. However, the travel time to health facilities was merely an estimate from self-reported data. Considering that two-thirds of the women had no formal education or at most primary education, there is a possibility of reporting errors for the travel time (minutes) to nearest health facility.

The findings from Cox regression revealed the contributory factors to distance as a barrier to reaching health facilities; these include geographical region, ethnicity and place of residence. This is consistent with report from a previous study [11]. In Senegal, geographical regions play a crucial role in healthcare delivery, influencing the distribution of resources and commodities to health facilities. Regions with better infrastructure typically enjoy more healthcare services and faster response times. However, less developed regions face significant burdens, with patients often traveling long distances to reach healthcare facilities [28]. The delay due to long travel time can be critical, especially in emergencies or disease management. The disparities in administrative efficiency and resource allocation can exacerbate issues. To mitigate this, strengthening regional administrative capacities and optimizing healthcare resource distribution are essential, ensuring timely access to healthcare services across all regions in Senegal. According to a previous study conducted in Nepal, there were regional differences in travel time to health facility, where about 80% of women in Karnali Province experienced delays in getting to a health facility for delivery, compared with 34% in Madhesh Province [29].

In this study, women from Dakar, Ziguinchor, Saint-Louis, Tambacounda, Kaolack, Thiès, Louga, Fatick, Kolda, Matam, Kaffrine, Kédougou, and Sédhiou reported a higher likelihood of distance as a barrier to reaching health facilities, when compared with women from Diourbel. Several of these regions are characterised by remote, rural areas with dispersed populations and limited road networks, increasing travel time or distance to healthcare facilities. In addition, regions with challenging terrains, such as Kédougou and Tambacounda have features that hinder easy accessibility. Diourbel is relatively smaller and more centralized, with better proximity to health facilities, reducing distance or travel time as barriers to reaching health facilities. Reducing long travel times to health facilities for women in Dakar and other urban regions is essential and requires targeted urban planning for healthcare need. Establishing strategically located health centers within densely populated neighbourhoods can minimize distance barriers. Strengthening public transportation networks, including affordable buses and dedicated health shuttles or ambulances, improves accessibility, especially for low-income women. Deploying mobile clinics and community health workers can extend services to underserved urban population. Integrating telemedicine solutions for consultations and follow-ups can reduce unnecessary travel. Policymakers should prioritise healthcare-responsive urban planning to address unique challenges faced by women, ensuring equitable access to timely and essential healthcare.

Women from Poular, Serer, and Mandingue ethnic groups had a lower risk of reporting distance as a barrier to health facilities, when compared with Wolof women. This can be due to differences in settlement patterns and community structures. Poular and Mandingue populations often inhabit rural areas with smaller, decentralized health facilities established to meet dispersed community needs. Serer communities have historically organized collective support systems, enabling easier access to transportation for health services. Conversely, Wolof women, predominantly residing in urban areas, may face challenges like traffic congestion and limited public transportation options, making travel to health facilities more time-consuming and distance as a barrier. Ethnicity plays a significant role in healthcare access and outcomes, intertwined with socio-cultural factors [30]. The country’s diverse ethnic groups, including the Wolof, Serer, and Pulaar, often have distinct living environments and socio-cultural statuses that could influence their healthcare utilization. Generally, ethnic groups residing in rural or remote areas typically face greater challenges in accessing healthcare facilities which may be due to the longer travel time or distances and inadequate transportation infrastructure [31]. The burden of distance is particularly acute for ethnic groups in certain regions, as the extended travel time to reach healthcare can lead to worsened health conditions. Improving transportation infrastructure and increasing the number of healthcare facilities in underserved areas are essential steps.

Rural women reported higher risk of reporting distance as a barrier in reaching a health facility. In Senegal, rural residents often facing greater challenges in distance to health facilities than urban dwellers. Remote locations typically have fewer healthcare facilities, resulting in longer distances and increased travel time. Furthermore, limited transportation infrastructure in rural areas can hinder timely access to care. The distance to reaching health facilities that provide emergency obstetric care, is most challenging in rural areas [7]. In some countries, living in remote and rural locations [32,33], characterised by poor road condition [25,33] delayed women from reaching care on time. Studies conducted in Gambia and in Nairobi slums [26,32] showed how the rainy season transforms roads into muddy pathways, with impossible driveability. In the rural Gambia [32], living next to a river meant being subject to floods which affected the availability of ferry services to reach the mainland and access care. In a number of studies [25,34], long travel time due to distance was cited among the main challenges to reach healthcare promptly. Improving access to healthcare through transport support programmes can reduce the barriers that prevent timely care. Investments in transportation infrastructure and the expansion of healthcare services in rural areas can enhance geographic accessibility.

## Strengths and limitations

This study utilised nationally representative data from the recent 2023 SDHS, making its findings of plausible comparison. The application of survival analysis in this context allows for the identification of factors that influence the travel time and distance as barrier to healthcare facility. By examining the time to event data, researchers can identify patterns and predictors of delays in seeking care. This information is crucial for designing targeted interventions that address the specific barriers faced by reproductive-aged women in accessing care in Senegal. There are two drawbacks to this analysis. First, the cross-sectional nature of the research findings makes it more difficult to determine the causal relationship between the independent and dependent variables. Secondly, travel time (minutes) to health facility was merely an estimate self-reported by women. Considering that two-thirds of the women had no formal education or at most primary education, there is a possibility of reporting errors for the travel time to nearest health facility. Therefore, caution must be taken in interpreting the travel time to health facility as reported by respondents.

## Conclusion

Based on the findings of this study, rural and remote women have distance as a barrier to healthcare facility. Travel time is a critical indicator of the burden of distance to a health facility and can therefore better inform health services planning for people residing in rural and remote locations in Senegal. Understanding the contributory factors to distance as a barrier in reaching a health facility is vital for improving maternal and child health outcomes. By addressing these factors through targeted interventions and policies, it is possible to reduce delays in seeking care and enhance health outcomes for women in Senegal. The government and stakeholders in healthcare sector should work to improve transportation infrastructure and provide mobile clinics to increase accessibility.

## Supporting information

S1 Fig Kaplan-Meier failure estimates of time to healthcare facility by age. (DOCX)

S2 Fig Kaplan-Meier failure estimates of time to healthcare facility by religion. (DOCX)

S3 Fig Kaplan-Meier failure estimates of time to healthcare facility by family motility. (DOCX)

S4 Fig Kaplan-Meier failure estimates of time to healthcare facility by education. (DOCX)

S5 Fig Kaplan-Meier failure estimates of time to healthcare facility by household wealth. (DOCX)

S6 Fig Kaplan-Meier failure estimates of time to healthcare facility by marital status. (DOCX)

S7 Fig Kaplan-Meier failure estimates of time to healthcare facility by visit to health facility within the previous 12 months. (DOCX)

S8 Fig Kaplan-Meier failure estimates of time to healthcare facility by health insurance coverage. (DOCX)
