# Supplementary figures and images for: Contributory factors to reporting distance as a barrier to health facility visit among reproductive-age Senegalese women: A survival analysis
Source: PLoS One. 2025 Apr 16;20(4):e0321850. doi: 10.1371/journal.pone.0321850 (PMC12002460; doi:10.1371/journal.pone.0321850)

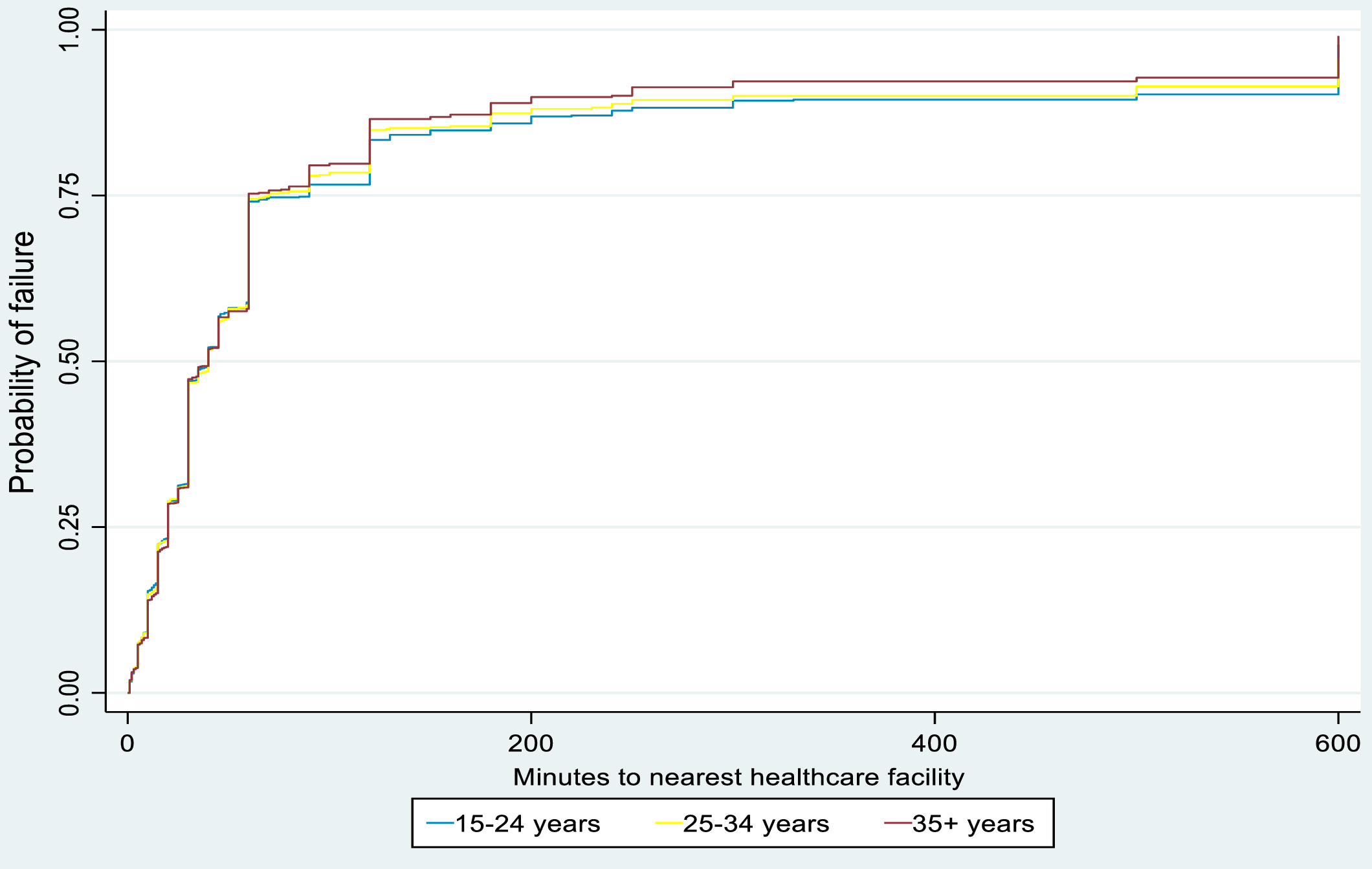


**Figure S1**. Kaplan-Meier failure estimates of time to healthcare facility by age

Supplement: S1 Fig — (DOCX) [file pone.0321850.s001.docx]

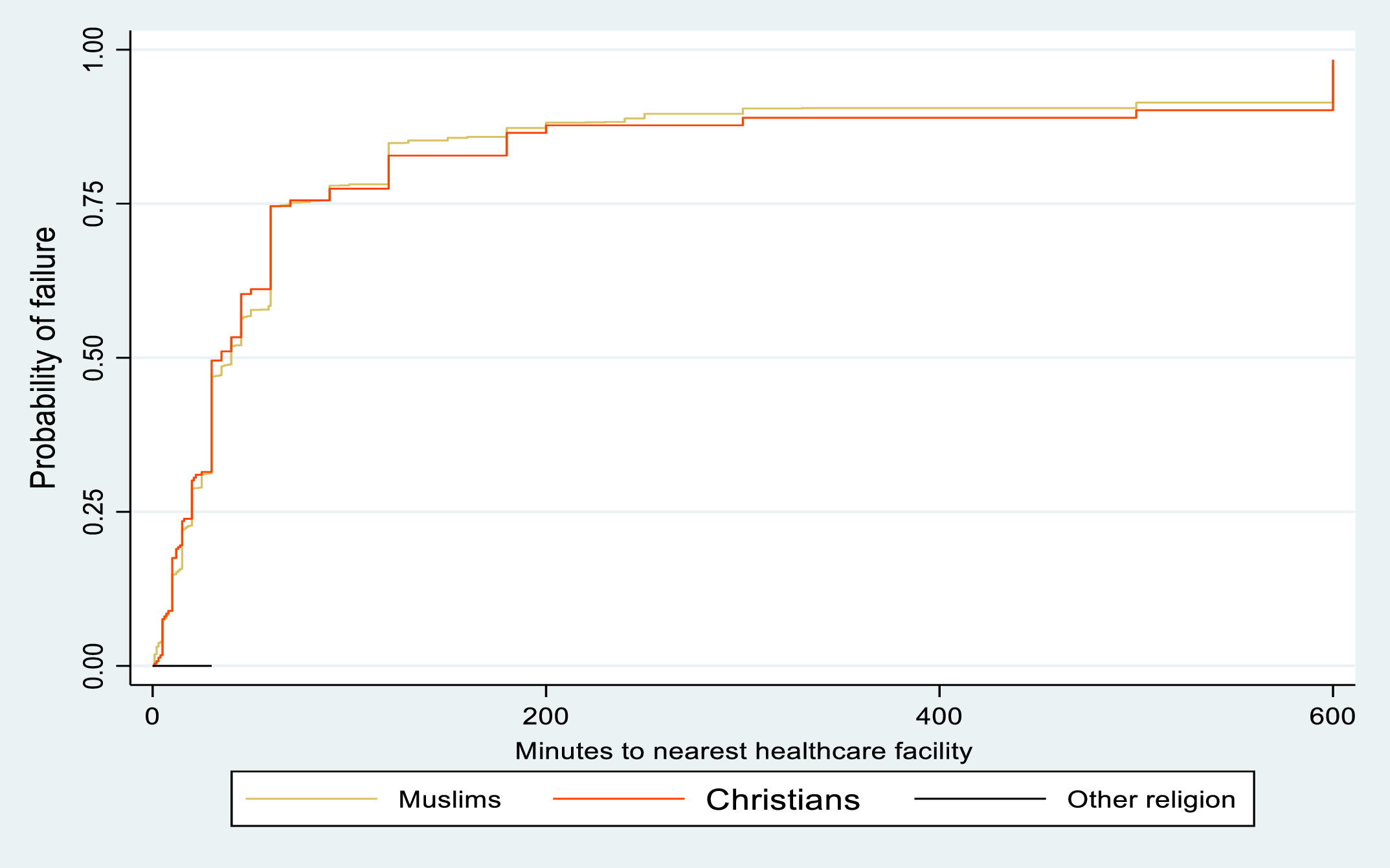


**Figure S2**. Kaplan-Meier failure estimates of time to healthcare facility by religion

Supplement: S2 Fig — (DOCX) [file pone.0321850.s002.docx]

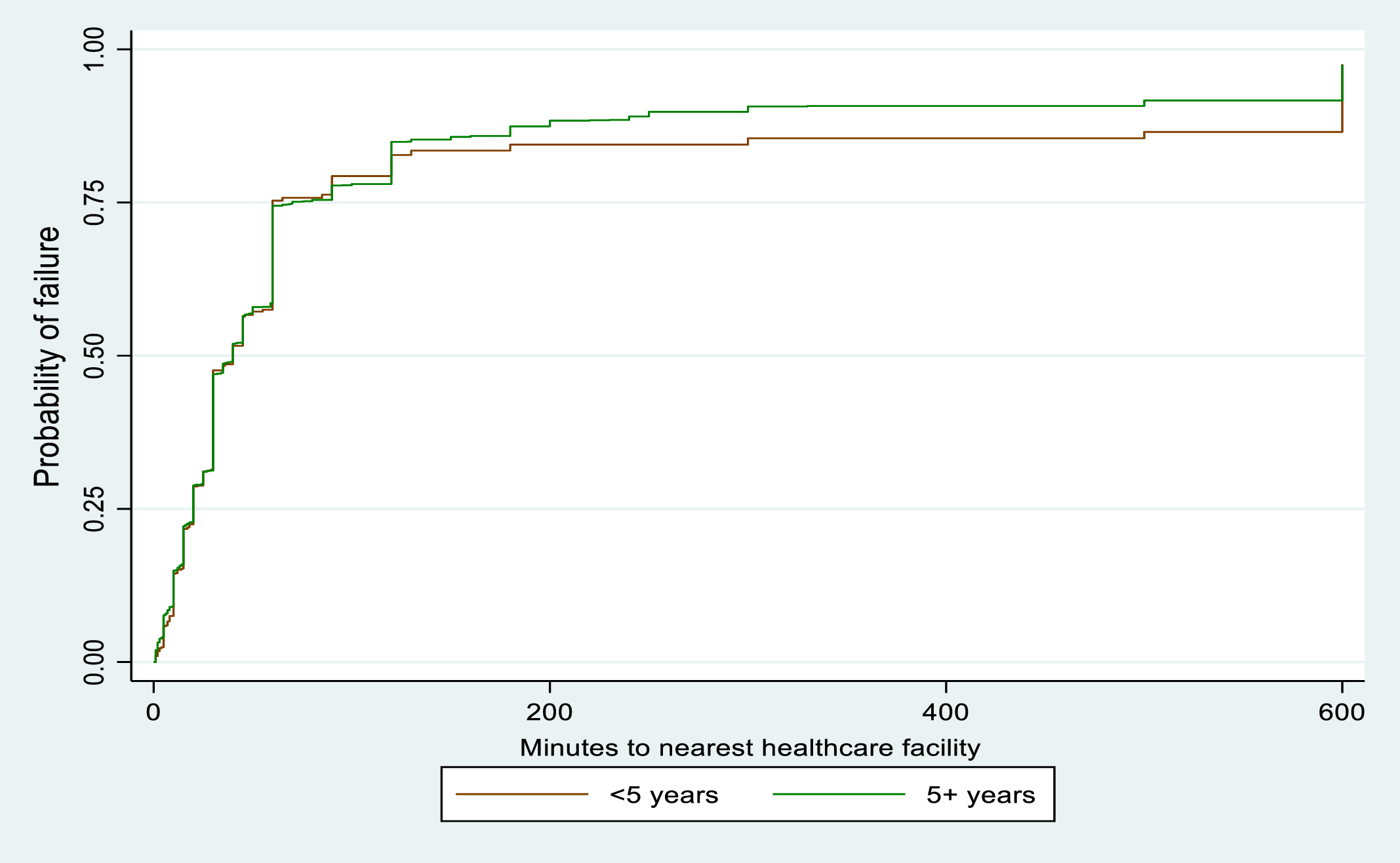


**Figure S3**. Kaplan-Meier failure estimates of time to healthcare facility by family motility

Supplement: S3 Fig — (DOCX) [file pone.0321850.s003.docx]

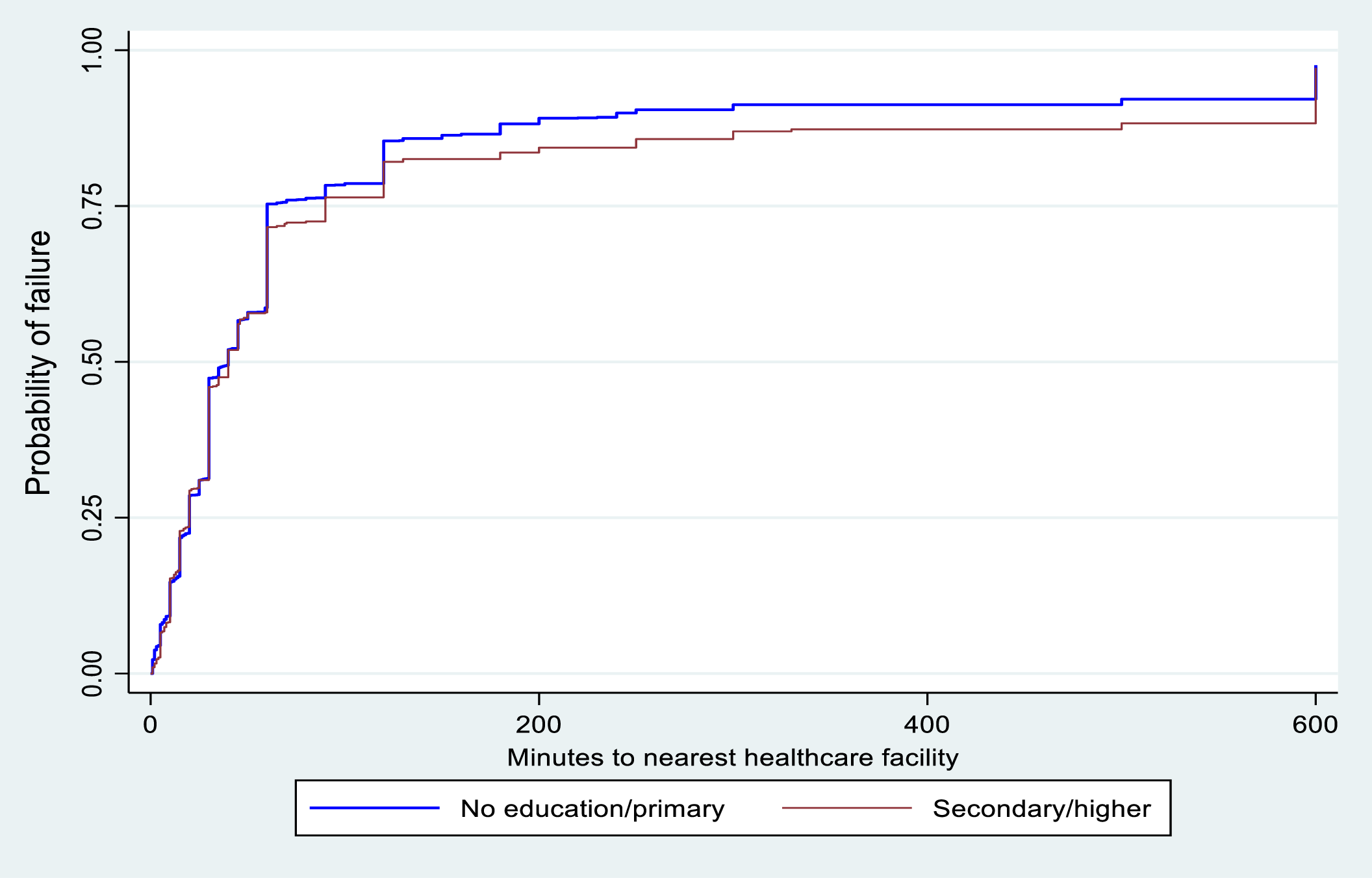


**Figure S4**. Kaplan-Meier failure estimates of time to healthcare facility by education

Supplement: S4 Fig — (DOCX) [file pone.0321850.s004.docx]

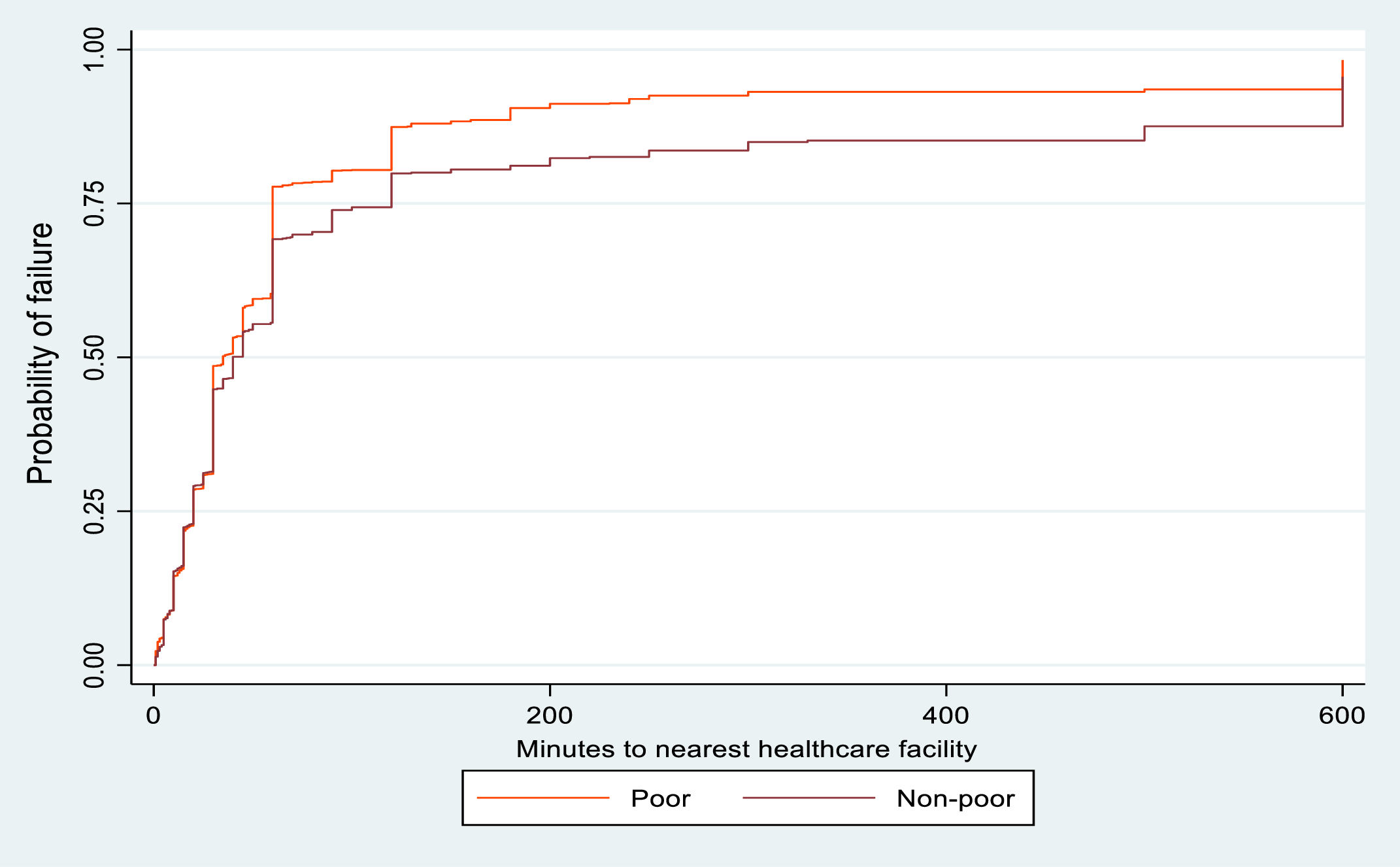


**Figure S5**. Kaplan-Meier failure estimates of time to healthcare facility by household wealth

Supplement: S5 Fig — (DOCX) [file pone.0321850.s005.docx]

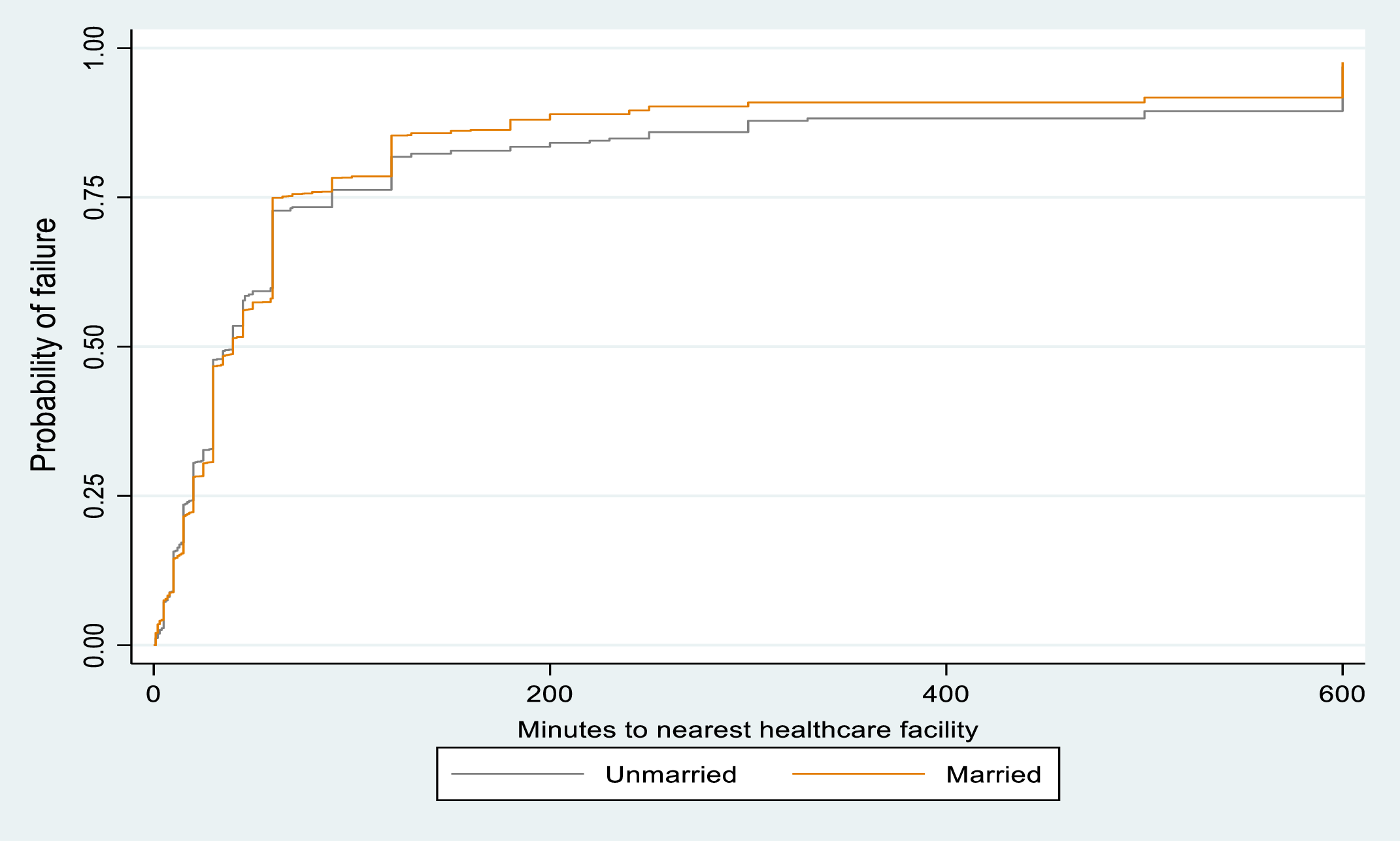


**Figure S6**. Kaplan-Meier failure estimates of time to healthcare facility by marital status

Supplement: S6 Fig — (DOCX) [file pone.0321850.s006.docx]
