# Supplementary material for: Contributory factors to reporting distance as a barrier to health facility visit among reproductive-age Senegalese women: A survival analysis
Source: PLoS One. 2025 Apr 16;20(4):e0321850. doi: 10.1371/journal.pone.0321850 (PMC12002460; doi:10.1371/journal.pone.0321850)
Supplement: S7 Fig — (DOCX) [file pone.0321850.s007.docx]

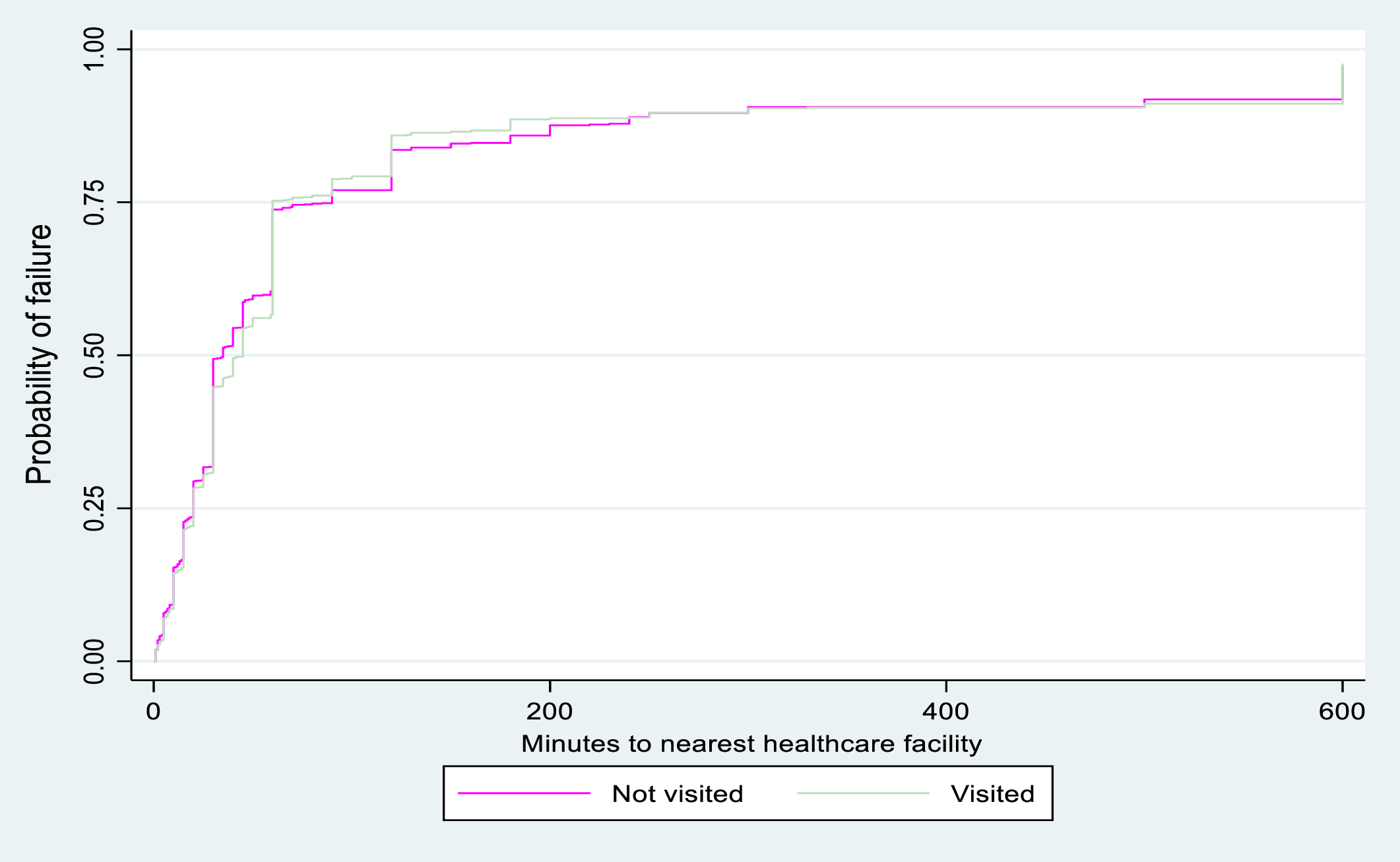


**Figure S7**. Kaplan-Meier failure estimates of time to healthcare facility by visit to health facility within the previous 12 months
